# Practical Chemistry and Pharmacy

**Published:** 1895-02

**Authors:** 

**Affiliations:** LaGrange, Ga.


### PRACTICAL CHEMISTRY AND PHARMACY.

EDITED BY HENRY R. SLACK, M.D., PH.M., LAGRANGE, GA.

### WEIGHTS AND MEASURES.

The following rules for the conversion of ordinary weights and measures into metric weights and measures will be found useful in practice and in the reading of medical and pharmaceutical works or papers.—(*The American Therapist*):

#### WEIGHT EQUIVALENTS.

To convert grains into grammes multiply by 0.065.

To convert grammes into grains multiply by 15.5.

To convert drachms into grammes multiply by 3.9.

To convert ounces (avoir.) into grammes multiply by 28.4.

To convert pounds (avoir.) into grammes multiply by 453.6.

#### MEASURE EQUIVALENTS.

To convert cubic centimeters into grains multiply by 15.5.

To convert cubic centimeters into drachms multiply by 0.26.

To convert cubic centimeters into ounces (avoir.) multiply by 0.036.

To convert pints into cubic centimeters multiply by 473.

To convert liters into ounces (avoir.) multiply by 35.3.

To convert gallons into liters multiply by 3.8.

---

### CREOSOTE PER RECTUM IN PHTHISIS.

Dr. Lemanski enumerates the advantages of the creosote suppository,—rapidity of absorption (equal to that of an enema), ease of administration, assured tolerance, and accurate and convenient dosage. He employed the following formula:

| | |
|---|---|
| Creosote (Beech) | 0.5 to 1 gm. |
| Cocoa butter | 5 gm. |

This method is said to possess the advantage of never disturbing the intestine and of always being readily accepted by the patients.

Dr. Simon has also taken up this subject (Thesis et Paris, 1894), being satisfied that, of all the modes of administration of this medicament, the rectal way is the simplest and best. He, too, refers to the rectal tolerance for creosote, to its ready absorption (proved by its elimination with the urine), and to numerous clinical facts testifying to the rapid amelioration of non-febrile tuberculous patients.

Simon's formula is this:

| | |
|---|---|
| Creosote (Beech) | xl. ctg. |
| Iodoform | xl. mg. |
| Salol | xl. ctg. |

This quantity is calculated for every ten kilogrammes of body weight, and is to be dissolved in nine parts of olive oil and injected per rectum once daily.—*Merck's Mark. Rep.*

---

### PRESERVATION OF SUBLIMATE SOLUTION.

L. Vignon, continuing his work on this subject, points out that the decomposition of sublimate solution is principally due to alkaline substances in the water employed or in the glass of which the recipients are formed, a limited quantity of such alkaline matter sufficing to cause the precipitation of a relatively considerable amount of mercury. On the other hand, hydrochloric acid and alkaline chlorides increase the stability of such solution, the first by saturating the alkaline precipitants, and the chlorides by their solvent power. As the result of a series of experiments, he finds that ammonium chloride prevents precipitation by ammonia or albuminoid matter in the water, but fails to prevent the action of sodium or sodium carbonate. Sodium chloride, on the other hand, fails in the case of ammonia and soda, but prevents precipitation by sodium carbonate and albumin. By combining the chlorides of ammonia and sodium, therefore, precipitation by any of the substances mentioned is prevented, as well as by hydrochloric acid. The two formulas recommended are as follows: (1) Mercuric chloride, 1 gm.; ammonium chloride, 20 gm.; sodium chloride, 10 gm.; distilled water, 1 liter. (2) Mercuric chloride, 1 gm.; hydrochloric acid (at 22° Baum.), 1 c. c.; distilled water, 1 liter.—*Western Druggist.*

### A DELICATE TEST FOR ALBUMIN IN THE URINE.

Dr. F. Spiegler recommends the following reagent, in testing for albumin in the urine:

| | | |
|---|---|---|
| ℞ | Distilled water | ℥ vjss. |
| | Corrosive sublimate | ʒ ij. |
| | Tartaric acid | ʒ j. |
| | Cane sugar | ʒ v. |

Some of the reagent is poured into a test-tube and the urine is added, little by little, after previously being filtered and acidulated, taking care that the two fluids do not mix. If it contains albumin, there appears at the point of contact a white precipitate at the zone of separation. This reagent will detect one part of albumin in one hundred and fifty parts of urine.—*Medical and Surgical Reporter.*

### TO DEODORIZE IODOFORM, CREOSOTE, AND GUAIACOL.

Attention is called by a German dermatological journal to the fact that the odor of iodoform, creosote, or guaiacol upon the hands can be overcome by washing with linseed meal. Articles having an odor of iodoform may be washed in tar-water to which oil of wintergreen has been added. The taste of pills of creosote can be disguised by means of a little coffee. The odor of iodoform or guaiacol in rooms can be dissipated by burning coffee.—*Western Druggist.*

We have tried this, and find it the best method of destroying the odor of iodoform we have ever used. H. R. S.

### REMOVAL OF RUST FROM STEEL INSTRUMENTS.

To remove rust from steel instruments Sangu recommends placing them over night in a salt-water solution of zinc chloride. The rust disappears through reduction. On removing the instruments they are to be rinsed with clear water, placed in a hot soda and soap solution, and dried. It is also advantageous to polish with absolute alcohol and chalk.—*Drug Circular.*

